# Facile Synthesis of Core-Shell Structured SiO_2_@Carbon Composite Nanorods for High-Performance Lithium-Ion Batteries

**DOI:** 10.3390/nano10030513

**Published:** 2020-03-12

**Authors:** Haibo Pang, Weicai Zhang, Peifeng Yu, Ning Pan, Hang Hu, Mingtao Zheng, Yong Xiao, Yingliang Liu, Yeru Liang

**Affiliations:** 1 College of Materials and Energy, Guangdong Provincial Engineering Technology Research Center for Optical Agriculture, South China Agricultural University, Guangzhou 510642, Chinamtzheng@scau.edu.cn (M.Z.); xiaoy@scau.edu.cn (Y.X.); 2 Guangdong Laboratory of Lingnan Mordern Agriculture, Guangzhou 510642, China

**Keywords:** SiO_2_, core-shell structures, composite, lithium-ion battery

## Abstract

Recently, SiO_2_ has attracted wide attention in lithium-ion batteries owing to its high theoretical capacity and low cost. However, the utilization of SiO_2_ is impeded by the enormous volume expansion and low electric conductivity. Although constructing SiO_2_/carbon composite can significantly enhance the electrochemical performance, the skillful preparation of the well-defined SiO_2_/carbon composite is still a remaining challenge. Here, a facile strategy of in situ coating of polydopamine is applied to synthesis of a series of core-shell structured SiO_2_@carbon composite nanorods with different thicknesses of carbon shells. The carbon shell uniformly coated on the surface of SiO_2_ nanorods significantly suppresses the volume expansion to some extent, as well as improves the electric conductivity of SiO_2_. Therefore, the composite nanorods exhibit a remarkable electrochemical performance as the electrode materials of lithium-ion batteries. For instance, a high and stable reversible capacity at a current density of 100 mA g^−1^ reaches 690 mAh g^−1^ and a capacity of 344.9 mAh g^−1^ can be achieved even at the high current density of 1000 mA g^−1^. In addition, excellent capacity retention reaches 95% over 100 cycles. These SiO_2_@carbon composite nanorods with decent electrochemical performances hold great potential for applications in lithium-ion batteries.

## 1. Introduction

Lithium-ion batteries (LIBs) have been widely used in portable devices, vehicle mounted equipment, and electrochemical energy storage owning to their environmental friendliness, high energy density, and long lifespan [1,2,3,4,5]. As the commercial anode material in LIBs, graphite has a low theoretical capacity of 372 mAh g^−1^, which is difficult to meet the ever-growing demands of high-energy-density [6,7,8]. Therefore, momentous efforts have been made to exploring an ideal alternative anode with high lithium storage capacity. Recently, SiO_2_ has attracted great attention for LIBs due to its low cost, high theoretical capacity (i.e., 1956 mAh g^−1^), and ease of fabrication [9,10,11]. Unfortunately, the practical application SiO_2_ anode is restricted by the fast capacity fading and poor rate performance, which mainly derive from its low electrical conductivity and huge volume expansion during the charging-discharging processes [12,13,14,15].

In order to circumvent these intrinsic limitations, two mainstream strategies have been proposed, i.e., design of nanostructures and compositing with high conductive carbon or other materials. For the former one, various nanostructured SiO_2_ such as nanofilms [16], nanocubes [17], nanospheres [18,19], nanotubes [20], and nanoparticles [21] have been successfully synthesized. This strategy greatly shortens the lithium ion and electron transport paths and increases the contact area between electrodes and electrolytes, and thus improves the electrochemical performance. However, the rate capability performance is still unsatisfactory because of the poor conductivity of SiO_2_ [22]. Compositing with high conductive carbon materials has been demonstrated to be a promising strategy to improve the electron transport and facilitate the electrochemical reaction kinetics, and thus enhance the rate performance [23,24]. In addition, the carbonaceous materials with a rational nanostructure can provide valuable void space to buffer a large volume expansion of SiO_2_, thus increasing the structural integrity and long lifespan of the active material [25,26,27]. Nevertheless, the facile preparation of well-defined SiO_2_/carbon composite anode is still full of challenge. Most of the general methods have various limitations, including rigid reaction conditions, complex multiple synthetic steps, and time-consuming. Moreover, control of the uniform carbon/SiO_2_ interface remains challenging.

**Scheme 1:**
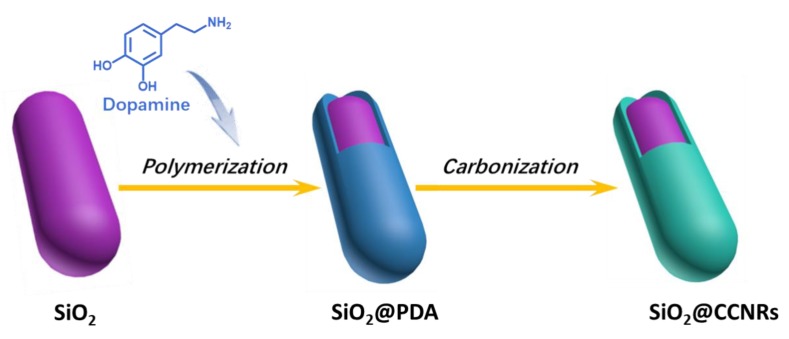
Schematic illustration of synthesis of the core-shell structured SiO_2_@CCNRs.

In this contribution, we report a facile method to prepare SiO_2_@carbon composite nanorods (SiO_2_@CCNRs) with a core-shell structure based on the in situ coating of polydopamine (PDA), demonstrating its superior ability when it is used as the anode material in LIBs. The synthetic process is exhibited in Scheme 1. PDA with a strong adhesive capability allows easy and quick construction of polymeric coating on the SiO_2_ nanorods. In addition, the polymerization of dopamine is executed in an aqueous solution at ambient temperature, which can provide a very mild and environmentally friendly technique. Moreover, by adjusting the amount of dopamine, it is easy to control the thickness of the PDA coating and the derivative carbonaceous shells of the SiO_2_@CCNRs. The SiO_2_@CCNRs composite architecture with the synergistic effect between highly active SiO_2_ core and conductive carbonaceous shell makes a prominent improvement to the excellent electrochemical performance. When evaluated as anode materials for LIBs, the as-synthesized SiO_2_@CCNRs with core-shell structure show excellent performance, such as a high and stable reversible capacity of 690 mAh g^−1^ after 100 cycles at 100 mA g^−1^, and a superior rate capability of 344.9 mAh g^−1^ even at the high current of 1000 mA g^−1^.

## 2. Materials and Methods

### 2.1. Materials

Triblock copolymer F127 was purchased from Sigma-Aldrich (Shanghai) trading Co., Ltd (Shanghai, China). Cetyltrimethylamine bromide (CTAB), tris-hydrochloride buffer (Tris-HCl buffer), and dopamine were purchased from Aladdin Biochemical Technology Co., Ltd (Shanghai, China). Aqueous ammonia (NH_3_·H_2_O) and tetraethyl orthosilicate (TEOS) were purchased from Sinopharm Chemical Reagent Co., Ltd (Shanghai, China). All chemical reagents were used as received without further purification.

### 2.2. Preparation of SiO_2_@Carbon Nanorods

#### 2.2.1. Synthesis of SiO_2_ Nanorods

Typically, 0.123 g of triblock copolymer F127 was added in the mixture of 3.50 mL of deionized water and 12.50 mL of the CTAB bromide solution and stirred at room temperature. Subsequently, 15 mL of NH_3_·H_2_O and 0.6 mL tetraethyl orthosilicate were added in the above solution under stirring. After quiescing for 4 h, the product was gathered by centrifuging and washed with distilled water until the filtrates was close to neutral. Finally, the SiO_2_ nanorods were obtained after drying.

#### 2.2.2. Synthesis of SiO_2_@Carbon Nanorods

In a typical process, 0.25 g of SiO_2_ nanorods were dispersed in 330 mL of Tris-HCl buffer solution by ultrasonic dispersion. After that, 0.25 g of dopamine was added to the above solution under stirring. After 24 h of stirring reaction, the outcome was gathered by centrifuging and then washed with distilled water several times and dried at 105 °C. The SiO_2_@CCNRs-1 was obtained after carbonizing at 900 °C for 6 h in the N_2_ atmosphere. The preparation processes of SiO_2_@CCNRs-2 and SiO_2_@CCNRs-3 were similar to the SiO_2_@CCNRs-1 except for the ratio of dopamine and SiO_2_ nanorods was 2 and 3, respectively.

### 2.3. Material Characterization

The composition of all the samples was determined by powder X-ray diffractometer (XRD, Rigaku Ultima IV, Tokyo, Japan) with Cu Kα radiation. The scanning electron microscope (SEM, FEI Quanta FEG650, Hillsboro, OR, USA) was used for testing the morphology of all the samples. The diameter of all the samples was determined from the particle size distribution by using the Nano Measurer analysis software through 100 nanorods randomly selected in the SEM image. The maximum in the resulting particle size distribution curve was referred to as the diameter of nanorods. The pore structure of all the samples was obtained using an ASAP 3Flex automatic volumetric sorption analyzer (Micromeritics sorption analyzer, Norcross, GA, USA) at 77 K. Moreover, the Brunauer–Emmett–Teller (BET) method was used to determine the specific surface area and total pore volume was calculated at a saturation relative pressure of ca. 0.99. The density functional theory (DFT) method was applied to analyze the pore size distribution.

### 2.4. Electrochemical Measurements

The electrode is mainly composed of active material (SiO_2_@CCNRs), conductive agent (Super-P), and binder (PVDF), with a mass ratio of 8:1:1. The slurry is coated on the copper foil, which is completely dried and cut into a circular piece with an area of 1.13 cm^2^. The average loading mass of each electrode piece is about 3.5 mg cm^−2^. Then, the CR2032 coin-type cells were assembled in the Ar-filled glove box, with lithium foil as the counter electrode. The electrolyte was 1 M LiPF_6_ in a 1:1:1 *v*/*v*/*v* mixture of ethylene carbonate (EC), methyl ethyl carbonate (EMC), and dimethyl carbonate (DMC). Galvanostatic charge-discharge tests were carried out in the range of 0.01 to 3.0 V on the Neware battery tester (Neware BTS 7.6×, Shenzhen, China). The specific capacity is calculated by dividing the tested capacity with the mass of active material (i.e., SiO_2_@CCNRs) in the battery. Cyclic voltammogram (CV) measurements data were recorded on the electrochemical workstation (Chenhua CHI660e, Shanghai, China) at a scan rate of 0.1 mV s^−1^ between 0.01 and 3 V. Electrochemical impedance spectroscopy (EIS) with the frequency range from 100 kHz to 10 mHz and an amplitude of 5 mV s^−1^ were carried out on a photoelectrochemical test system (Modulab XM DSSC, Bognor Regis, UK).

## 3. Results

**Figure 1:**
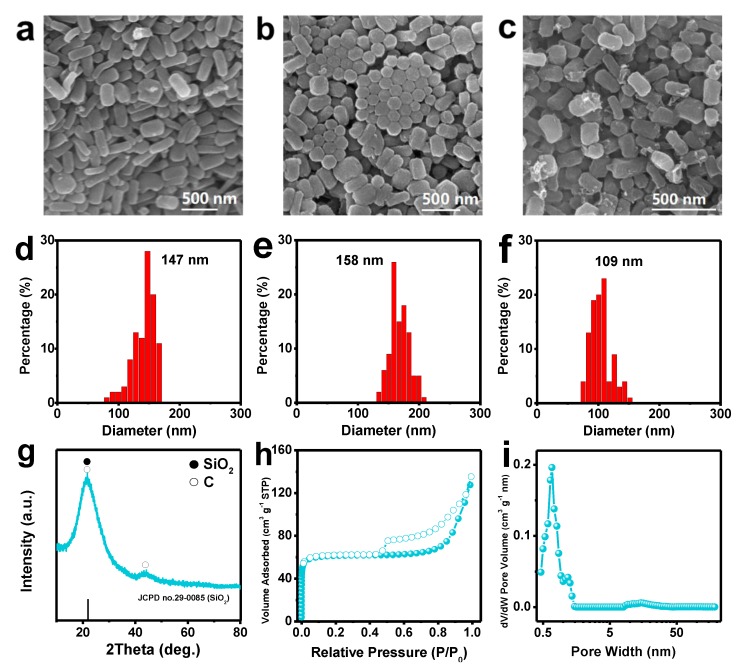
SEM images of (**a**) SiO_2_, (**b**) SiO_2_@PDA-1 complex, and (**c**) SiO_2_@CCNRs-1. Particle size distributions of (**d**) SiO_2_, (**e**) SiO_2_@PDA-1, and (**f**) SiO_2_@CCNRs-1. (**g**) XRD pattern, (**h**) N_2_ adsorption-desorption isotherm, and (**i**) pore size distribution curve of SiO_2_@CCNRs-1.

The fabrication procedure of SiO_2_@CCNRs is schematically illustrated in Scheme 1. Uniform SiO_2_ nanorods with an average diameter of 147 nm were first synthesized (Figure 1a,d). Subsequently, dopamine was polymerized in an aqueous solution at room temperature to form PDA with strong adhesion ability, which can easily and quickly in situ construct a polymer coating on SiO_2_ nanorods. The obtained SiO_2_@PDA-1 with about 5.5 nm thicknesses of PDA coating reveals the nanorod structure consistent with SiO_2_ (Figure 1b,e). After carbonization under nitrogen protection, the targeted SiO_2_@CCNRs were obtained. As shown in Figure 1c, the SiO_2_@CCNRs-1 still maintain a relatively good nanorods morphology, meaning good structural inheritance after carbonization. The average diameter of the SiO_2_@CCNRs-1 is 109 nm (Figure 1f), which is about 49 nm smaller than that of SiO_2_@PDA-1. This could be due to the shrinkage of carbon and SiO_2_ caused by mass loss after carbonization. By changing the amount of dopamine, the thickness of the carbon shell on the SiO_2_ nanorods can be simply controlled. For example, by increasing the use of dopamine by 1 and 2 times, the average diameters of obtained SiO_2_@CCNRs-2 and SiO_2_@CCNRs-3 are increased by 5 and 10 nm compared to SiO_2_@CCNRs-1, respectively (Figure S1). This indicates that the thickness of carbon shell increases with the increase of dopamine content.

XRD is conducted to study the microcrystalline structure of SiO_2_@CCNRs. Figure 1g and Figure S2 show the XRD patterns of the SiO_2_@CCNRs. The peaks located at 22° indexed to SiO_2_ (JCPD Card No. 29-0085), and the broad peaks located at 23° is associated with carbon, while the weak peak near 44° can be attributed to amorphous carbon [28,29,30]. To explore the pore characteristic of the SiO_2_@CCNRs, the N_2_ adsorption-desorption measurement was executed. As shown in Figure 1h, the N_2_ adsorption-desorption isotherm shows a typical type IV isotherm, which shows that at a lower relative pressure (P/P_0_), the adsorption amount of N_2_ increases rapidly, and a hysteresis loop appears in the range of 0.4 to 1.0, demonstrating the presence of the micropores and mesoporous [31]. It can be seen from the pore size distribution curve (Figure 1i) that the pore size of the micropores of SiO_2_@CCNRs-1 mainly exists at 0.68 nm, and the mesopores ranges from 8.6 to 29.5 nm. In addition, the total pore volume and BET surface area of SiO_2_@CCNRs-1 is 0.17 cm^3^ g^−1^ and 246 m^2^ g^−1^.

**Figure 2:**
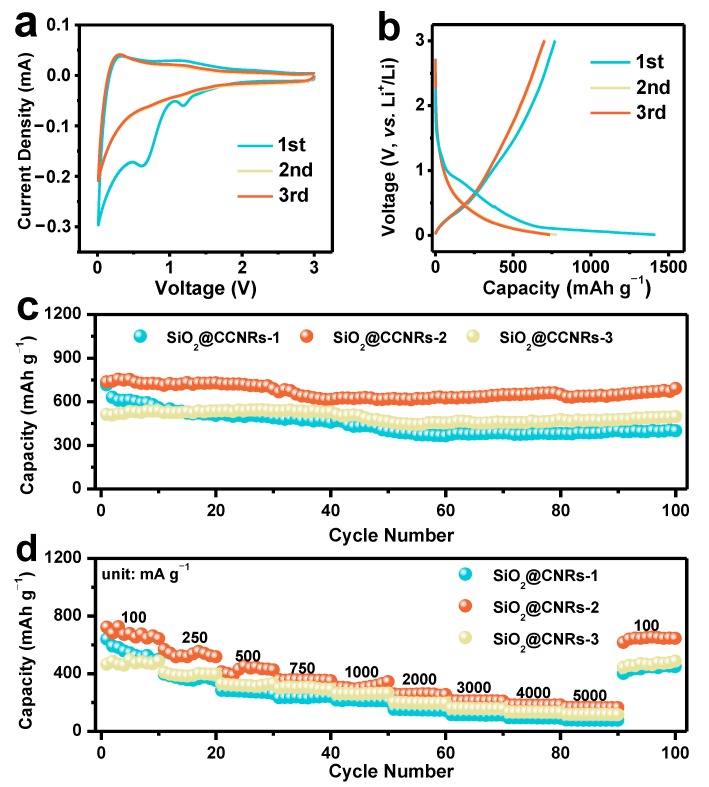
(**a**) Cyclic voltammogram (CV) curves of SiO_2_@CCNRs-2 cycled between 0.01 and 3.0 V (vs. Li^+^/Li) at a scanning rate of 0.1 mV s^−1^ for the first three cycles. (**b**) Galvanostatic discharge/charge curves of SiO_2_@CCNRs-2 electrode at current density of 100 mA g^−1^ for the first three cycles. (**c**) Comparison of the cycling performance of the SiO_2_@CCNRs electrode at a current density of 100 mA g^−1^ for 100 cycles. (**d**) Rate performance of the SiO_2_@CCNRs electrode at different current densities.

The lithium ion storage behavior of SiO_2_@CCNRs as anode material for LIBs is explored. Figure 2a shows representative CV curves for the SiO_2_@CCNRs-2 electrode at a scanning rate of 0.1 mV s^−1^ between 0.01 and 3.0 V. In the first cycle, an obvious reduction peak is observed at ~0.7 and ~1.1 V but disappears in the subsequent two cycles, which was related to formation of the solid-electrolyte-interface (SEI) layer and electrolyte decomposition on the surface of the carbon shell [32,33]. In the second and third cycles, a weak cathodic peak appears at about 0.3 V, corresponding to the formation of Li-Si alloy, while the broad anodic peak at 0.5–1.7 V is assigned to the dealloying processes of Li-Si alloy into Si [34]. Furthermore, the second and third CV curves overlap well, demonstrating the reversible alloying/dealloying reaction [35]. Figure 2b shows that there are two voltage plateaus at ~0.7 and ~1.1 V in the initial discharge curve, which is consistent with the results from the CV curve. The voltage plateaus of the later discharge curves are in the range of 0.01–0.5 V.

The charge/discharge cycling performances of SiO_2_@CCNRs are displayed in Figure 2c. They show a stable cycling stability up to 100 cycles of charge/discharge at a current density of 100 mA g^−1^. It can be clearly seen that SiO_2_@CCNRs-2 with moderate carbon shell thickness delivers a stable reversible specific capacity at about 690 mAh g^−1^ after 100 cycles, which is much higher than that of SiO_2_@CCNRs-1 (i.e., 423 mAh g^−1^) and SiO_2_@CCNRs-3 (i.e., 498 mAh g^−1^). The degradation of the cycle stability for SiO_2_@CCNRs-1 could be attributed to the peeling behavior of the thin carbon layer caused by the volume change of SiO_2_. The rate performances of the SiO_2_@CCNRs anode are further tested at different current densities from 100 to 5000 mA g^−1^ (Figure 2d). At current densities of 100, 250, 500, 750, 1000, 2000, 3000, 4000, 5000 mA g^−1^, the reversible specific capacities of the SiO_2_@CCNRs-2 anode are ~730, 573, 464, 362, 315, 267, 213, 187, and 165 mAh g^−1^, respectively. Furthermore, when the current density is returned to 100 mA g^−1^, SiO_2_@CCNRs-2 delivers high reversible specific capacity at about 645 mAh g^−1^, about 95% of the initial specific capacity could be recovered. Similarly, SiO_2_@CCNRs-1 and SiO_2_@CCNRs-3 also show good rate performance, demonstrating the superiority of this type of composite material.

**Figure 3:**
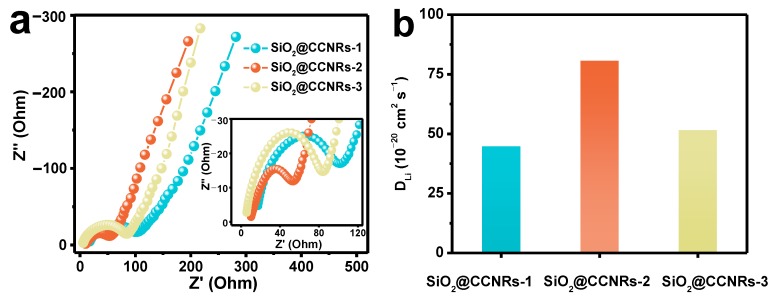
(**a**) Nyquist plots and (**b**) lithium ion diffusion coefficients of different anodes inLIBs.

The reaction kinetics and electrochemical activities of electrodes is studied by electrochemical impedance spectroscopy (EIS). Figure 3a and Figure S3 show the Nyquist plots and plots of Z’ against ω^−1/2^, respectively. The relevant kinetic data are calculated and summarized in Table 1. It can be seen that the charge transfer resistance (R_ct_) of SiO_2_@CCNRs-2 is 43.14 Ω, which is much lower than that of SiO_2_@CCNRs-1 (i.e., 83.64 Ω) and SiO_2_@CCNRs-3 (i.e., 78.26 Ω), indicating that SiO_2_@CCNRs-2 has a higher charge transfer efficiency. Figure 3b shows the lithium diffusion ion coefficients of different anodes in LIBs. The lithium ion diffusion coefficient of SiO_2_@CCNRs-2 is calculated to be 8.09 × 10^−19^ cm^2^ s^−1^, which is higher than that of SiO_2_@CCNRs-1 (4.49 × 10^−19^ cm^2^ s^−1^) and SiO_2_@CCNRs-3 (5.18 × 10^−19^ cm^2^ s^−1^). It is illustrated that SiO_2_@CCNRs-1 has a more rapid lithium ion diffusion rate.

From the above analysis and discussion, the SiO_2_@CCNRs-2 anode displays more excellent lithium ion diffusion rate than the SiO_2_@CCNRs-1 and SiO_2_@CCNRs-3 anodes, which mainly comes down to the moderate carbon shell thickness in SiO_2_@CCNRs. On the one hand, the thin carbon shell of SiO_2_@CCNRs-1 could not effectively ameliorate the rapid volume expansion of SiO_2_ during the cycles, and could not constantly inhibit the pulverization of electrode either, resulting in the increase of resistance and the decrease of lithium ion diffusion rate. On the other hand, the thick carbon shell of SiO_2_@CCNRs-3 increases the ion diffusion distance, which decreases the mass transfer efficiency. As a comparison, the moderate carbon shell thickness of SiO_2_@CCNRs-2 not only effectively relieves the volume expansion of SiO_2_ without cracking, but also maintains good kinetic characteristics, thus exhibiting more excellent electrochemical performance.

## 4. Conclusions

The core-shell structured SiO_2_@CCNRs composite was successfully synthesized based on the in situ coating of PDA. The carbon shell on the surface of SiO_2_ not only increases the conductivity of SiO_2_, but also reduces the volume change of SiO_2_ during charging and discharging. Therefore, the core-shell structured SiO_2_@CCNRs exhibit excellent lithium storage capacity, rate performance, and cycle stability. In view of this, the SiO_2_@CCNRs are a kind of high-performance LIBs electrode materials with excellent application potential. Moreover, the effect of carbon shell thickness on the electrochemical performance of LIBs is studied as well. It can be predicted that further optimization of electrochemical performance could be realized by tuning of the size of SiO_2_ nanorod, carbonization temperature, and heating rate. Meanwhile, it is envisioned that this strategy can be extended to those carbon precursors that have a strong adhesive capability to form homogeneous and robust organic/inorganic interfaces with SiO_2_, which can also provide an opportunity for further boosting their electrochemical performance as well. We hope that this work can provide new ideas for the efficient preparation of high-performance SiO_2_/carbon composite electrode materials, and help the rapid development of silicon-based electrode materials.

## Figures and Tables

**Table 1:** Kinetic parameters of SiO_2_@CCNRs-1, SiO_2_@CCNRs-2, and SiO_2_@CCNRs-3 anodes.

Key Parameters	SiO_2_@CCNRs-1	SiO_2_@CCNRs-2	SiO_2_@CCNRs-3
R_s_ (Ω)	17.42	10.22	5.69
R_ct_ (Ω)	83.64	43.14	78.26
σ (Ω Hz^1/2^)	191.89	142.92	178.5
D_Li_ (10^−19^ cm^2^ s^−1^)	4.49	8.09	5.18

## Supplementary Materials

The following are available online at https://www.mdpi.com/2079-4991/10/3/513/s1, Figure S1: SEM images of (a) SiO_2_@CCNRs-2 and (b) SiO_2_@CCNRs-3. Particle size distributions of (c) SiO_2_@CCNRs-2 and (d) SiO_2_@CCNRs-3; Figure S2: XRD patterns of SiO_2_@CCNRs-2 and SiO_2_@CCNRs-3; Figure S3: Plots of ω^−1/2^ versus Z’ of SiO_2_@CCNRs composites anodes in LIBs.
