# Supplementary material for: Facile Synthesis of Core-Shell Structured SiO2@Carbon Composite Nanorods for High-Performance Lithium-Ion Batteries
Source: Nanomaterials (Basel). 2020 Mar 12;10(3):513. doi: 10.3390/nano10030513 (PMC7153475; doi:10.3390/nano10030513)
Supplement: Supplementary file 1 [file nanomaterials-10-00513-s001.pdf]

Supplementary Materials:

Facile Synthesis of Core-Shell Structured SiO_2@Carbon Composite Nanorods for High-Performance Lithium-Ion Batteries

Haibo Pang [1,2,†], Weicai Zhang [1,2,†], Peifeng Yu [1,2,†], Ning Pan [1], Hang Hu [1,2,*], Mingtao Zheng [1,2], Yong Xiao [1,2], Yingliang Liu [1,2,*] and Yeru Liang [1,2,*]

[1] College of Materials and Energy, Guangdong Provincial Engineering Technology Research Center for Optical Agriculture, South China Agricultural University, Guangzhou 510642, China; 20183077050@stu.scau.edu.cn (H.P.); 20172008018@stu.scau.edu.cn (W.Z.); yupf@stu.scau.edu.cn (P.Y.); 201430260116@stu.scau.edu.cn (N.P.); mtzheng@scau.edu.cn (M.Z.); xiaoy@scau.edu.cn (Y.X.)

[2] Guangdong Laboratory of Lingnan Mordern Agriculture, Guangzhou 510642, China

* Correspondence: huhang@scau.edu.cn (H.H.); tliuyl@scau.edu.cn (Y.L.); liangyr@scau.edu.cn (Y.L.); Tel.: +86-020-8734-4806 (Y.L.); +86-020-8528-0319 (Y.L.)

† These authors contributed equally to this work.

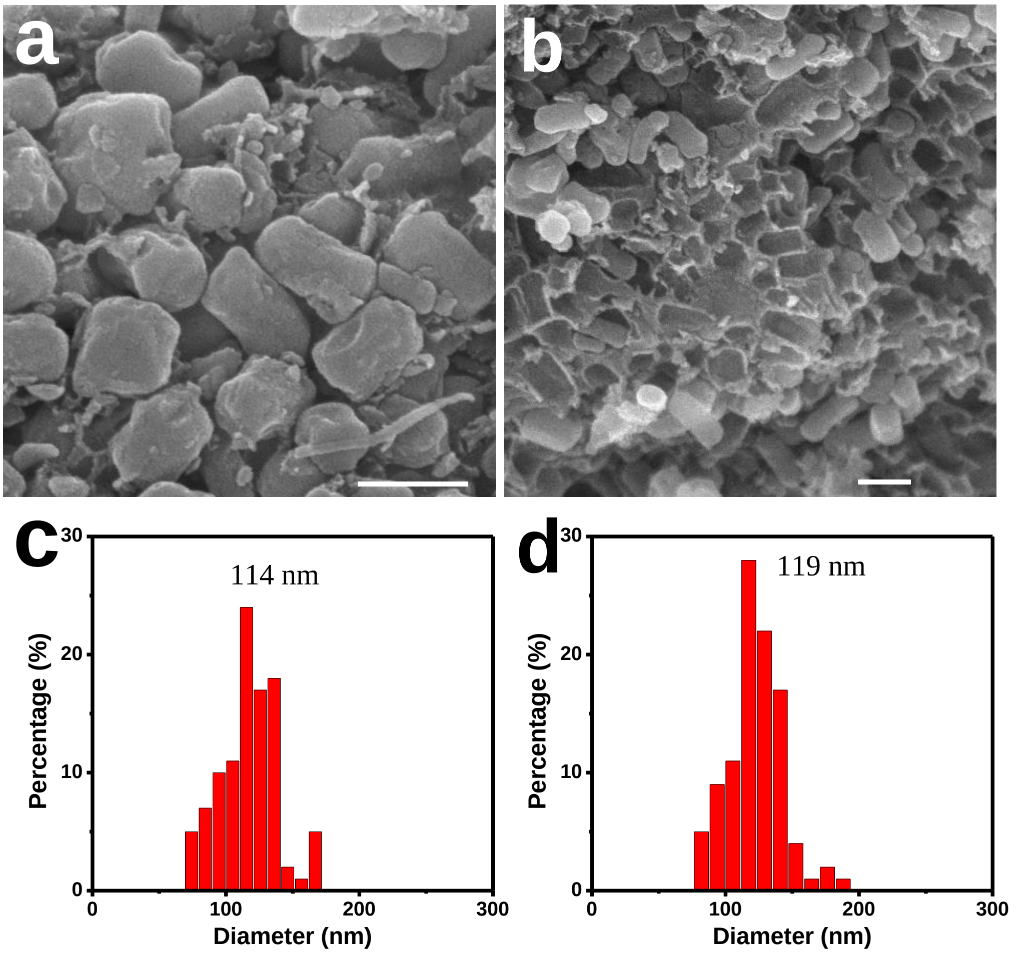

Figure S1. SEM images of (**a**) SiO_2@CCNRs-2, (**b**) SiO_2@CCNRs-3. Particle size distributions of (**c**) SiO_2@CCNRs-2, (**d**) SiO_2@CCNRs-3.

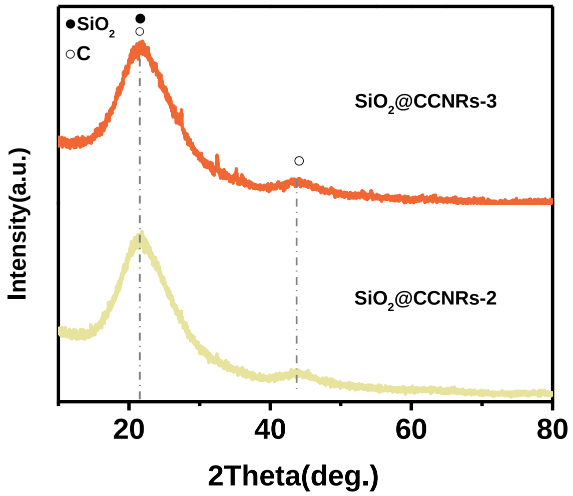

Figure S2. XRD patterns of SiO_2@CCNRs-2 and SiO_2@CCNRs-3.

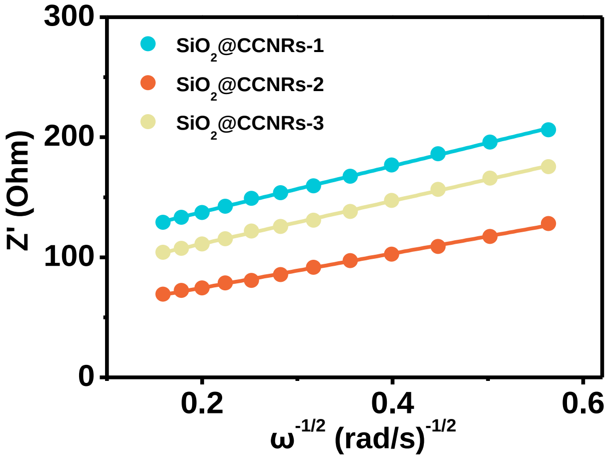

Figure S3. Plots of $\omega^{-1/2}$ versus Z′ of SiO_2@CCNRs composites anodes in LIBs.
